# Simulation-Based Analysis of “What-If” Scenarios with Connected and Automated Vehicles Navigating Roundabouts

**DOI:** 10.3390/s22176670

**Published:** 2022-09-03

**Authors:** Maria Luisa Tumminello, Elżbieta Macioszek, Anna Granà, Tullio Giuffrè

**Affiliations:** 1 Department of Engineering, University of Palermo, Viale delle Scienze ed 8, 90128 Palermo, Italy; 2 Department of Transport Systems, Traffic Engineering and Logistics, Faculty of Transport and Aviation Engineering, Silesian University of Technology, Krasińskiego 8 Street, 40-019 Katowice, Poland; 3 Faculty of Engineering and Architecture, University of Enna Kore, Viale della Cooperazione, 94100 Enna, Italy

**Keywords:** road infrastructure, roundabout, entry capacity, microscopic simulation, connected automated vehicles

## Abstract

Despite the potential of connected and automated vehicles (CAVs), there are still many open questions on how road capacity can be influenced and what methods can be used to assess its expected benefits in the progressive transition towards fully cooperative driving. This paper contributes to a better understanding of the benefits of CAV technologies by investigating mobility-related issues of automated vehicles operating with a cooperative adaptive cruise control system on roundabout efficiency using microscopic traffic simulation. The availability of the adjustment factors for CAVs provided by the 2022 Highway Capacity Manual allowed to adjust the entry capacity equations to reflect the presence of CAVs on roundabouts. Two mechanisms of entry maneuver based on the entry lane type were examined to compare the capacity target values with the simulated capacities. The microscopic traffic simulator Aimsun Next has been of great help in building the “what-if” traffic scenarios that we analysed to endorse hypothesis on the model parameters which affect the CAVs’ capabilities to increase roundabouts’ throughput. The results highlighted that the increasing penetration rates of CAVs have greater impacts on the operational performances of roundabouts, and provided a synthetic insight to assess the potential benefits of CAVs from an efficiency perspective.

## 1. Introduction

Whether connected and automated vehicles (CAVs as they are known) can negotiate efficiently or hesitate before entering the roundabouts is still an open issue [1,2,3]. The cooperative adaptive cruise control technology (CACC) for CAVs which combines the adaptive cruise control with the cooperative decision making enabled by vehicle-to-vehicle communication should bring novel opportunities to the digital community of road users concerning the exchange and sharing of data on the vehicles’ dynamic states, the driving intentions and the surrounding context [4,5]. The cooperative driving should enhance the car-following behaviour at roundabouts, thus improving the entry capacity, especially under balanced traffic flows from all entries [3,6,7,8]. Although the transition towards increasingly high levels of connected and automated transportation should resolve the variability in human performance, the priority rule to vehicles circulating around the central island and the predominantly curved trajectories of the roundabouts may make the decision making by vehicles instead of human drivers even more challenging than at stop-controlled or signalised intersections [9,10,11]. There is still the need to deepen the knowledge about high automation level or full dynamic navigation without human participation on roundabouts, also because CAVs capable of fully controlling the vehicle for a trip or under any operating condition are not yet in production for consumers [9,12,13]. Besides the issues on CAV management where the curvilinear geometric design prevails and breaks the continuity of the straight path [1], other open questions concern the users’ awareness of the best potential of CAVs in relation to the economic and environmental sustainability of the collaborative autonomous driving (e.g., [12,14]), the consumer attitudes towards the acceptability of CAVs in a transient state of their implementation (e.g., [15,16]), and the key road operational issues when human-driven vehicles (hereinafter HDVs) and CAVs are mixed in the same road or intersection at the same time (e.g., [17]). There are still low market penetration rates of CAVs on road segments, intersections or roundabouts, and it could take years to adapt the regulatory framework, the vehicles, the communication elements that the roads should already have in place and the whole system reliability to the new market requirements [18]. Thus, any analysis should be based on current knowledge and behavioural assumptions about the ability of CAVs to safely and efficiently operate on roundabouts. 

Data mining approaches and machine learning techniques have been already employed to assess the detection and navigation on roundabouts with and without surrounding traffic [19]. However, the expert system should manage a wide range of time-consuming procedures before achieving a very high success rate in the driving decision-making processes [20]. In recent years, an increasing number of studies focused on the computational efficiency of the microscopic traffic simulation models to analyse whether or not CAVs negotiate any road infrastructure as a human driver would do and they are able to improve traffic throughput, as expected (e.g., [21]). However, there are not enough studies that incorporate the input parameters uncertainty, particularly where assumptions related to CAVs cannot be calibrated to actual operating conditions [2]. Thus, driving simulation modelling using CAV logic should be applied to assess “what-if” traffic scenarios which should not be regarded as the last word on what will happen when CAVs will be widespread on the road network [2,22]. In this view, the adjustment factors for freeway elements, signalised intersections and roundabouts, recently presented by the Highway Capacity Manual (HCM) [2], are the only usable reference to determine the CAV-adjusted capacity relationships in heterogenous traffic. Given the absence of level 4 and 5 CAVs using the actual road infrastructures [9], the adjustment factors for CAVs were based on all the knowledge gained during the last years and were derived from microsimulation under the assumption that all communication elements required to provide their full cooperation were working with a high degree of reliability [2,3,23]. 

Based on the above, this paper presents a “what-if” scenarios analysis to assess mobility-related issues with CAVs on roundabouts using microscopic traffic simulation. The focus has been put on vehicles equipped with cooperative adaptive cruise control features that are able to accept shorter gaps than HDVs or automated vehicles (AVs), using adaptive cruise control only [12]. Since field measurements are still infeasible due to the current lack of significant proportions of CAVs in traffic, the CAV-adjusted capacity curves for roundabout systems by the HCM [2] were used as an alternative source of capacity target values to which the simulation output were then compared; see also [24]. It should be noted that the HCM [2] allows the analyst to determine the CAV-adjusted capacity values for different mechanisms of entry maneuver into roundabouts where the one-lane entry can be conflicted by one or two circulating lanes, or where each lane in a two-lane entry can be conflicted by one circulating lane or two circulating lanes. 

The paper investigated two mechanisms of entry that were based on empirical observations of actual vehicles on actual single-lane and two-lane roundabouts: the one-lane entry conflicted by one circulating lane—hereinafter entry mechanism (1); and the left entry lane in a two-lane entry conflicted by two circulating lanes—hereinafter entry mechanism (2). According to [2], for each entry mechanism, a fleet made only by HDS was employed to create the capacity curve of the baseline scenario, while the CAV-adjusted capacity curves were created for a percentage of CAVs in traffic varying from 20% to 100% in 20% increments. Operating conditions at capacity were then simulated in Aimsun Next [25] for the two mechanisms of entry which we examined. Specifically, the objectives of the research consisted in answering the following questions:

- How to model and to account for the interactions of CAVs with HDVs on roundabouts where operations at capacity are going to be reached?
- How to revise the values of the model parameters so as to improve the agreement of the simulated data with the capacity target values calculated under different proportions of CAVs in traffic?
- How to analyse the change in driving behaviour and how to assess the potential benefits of greater proportions of CAVs on roundabouts?

The microscopic traffic simulator Aimsun Next [25] has been of great help in building “what-if” traffic scenarios designed to endorse hypothesis on cooperative driving in mixed fleets of HDVs and CAVs, and to investigate the CAV effects on roundabout efficiency in the progressive transition towards an all-CAV fleet. Performance measures were selected to explain the impacts on roundabouts due to the technological change in vehicles, subject to there being no changes to the real-world road network that may invalidate our assumptions. With the awareness that any evaluation of traffic conditions on the examined roundabout layouts can be seen just as a potential outcome rather than being thought of as the final word on what may happen once CAVs will be fully operational on the road infrastructures, the main considerations of the study concern the feasibility of the fine-tuning process of the behavioural parameters of Aimsun Next [25] that, in turn, made the model valid to explain the mechanisms of entry into the roundabouts that we identified, and to answer the questions above. 

Although the results can be influenced by the assumptions concerning the CAV driving and gap usage, and the microscopic traffic simulator that we used, the results showed that the introduction of CAVs in traffic can provide a general performance improvement compared to the baseline scenario with HDS for both mechanisms of entry into the roundabouts here investigated. However, a final conclusion on the best geometric design of the roundabouts has not been possible to reach since more entry lane types or roundabouts of different sizes should be investigated. 

**Figure 1:**
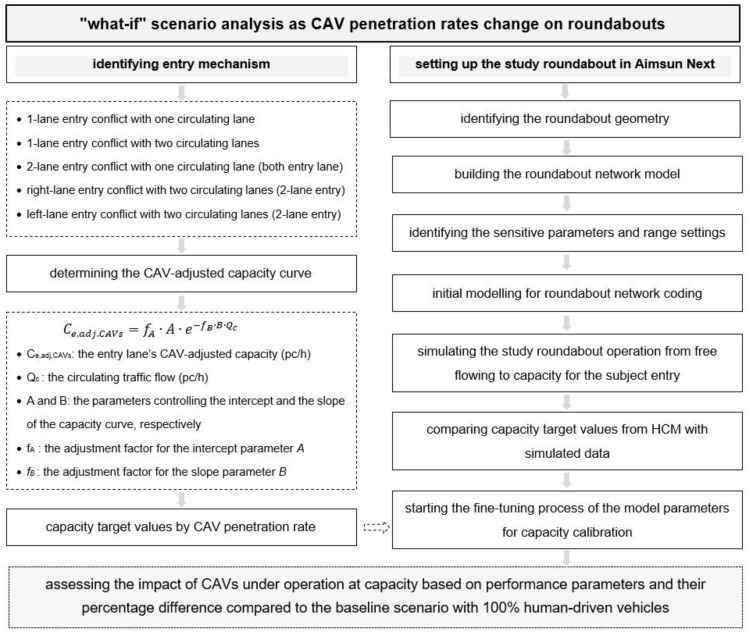
The summary framework of the proposed methodology. Source: Own research started from data presented in [2].

Based on the above, the scientific contribution of the research includes the analysis of the effects of the model parameters on the mechanisms of entry here examined and a synthetic insight to assess the potential benefits of the CAVs on roundabouts from an efficiency perspective. In turn, the societal contribution of the paper can be attributable to a greater understanding of the potential benefits of the CAV technologies to be implemented in traffic management strategies. Figure 1 shows the framework of the methodological path followed in this research. 

The articulation of the paper is as follows: Section 2 introduces some emerging issues and concerns on cooperative driving, the users’ attitudes toward the CAV acceptability and the latest research on microsimulation modelling with CAVs on roundabouts. Section 3 describes the steps we have taken in Aimsun Next [25] to model the presence of CAVs in the traffic stream and the fine-tuning process of the model parameters, while Section 4 presents the results of the analysis of the “what-if” scenarios under different market penetration rates of CAVs on roundabouts. In turn, Section 5 discusses the results, while Section 6 concludes the paper and shows the future developments of the research.

## 2. Literature Review and Related Research

This section introduces emerging issues on cooperative driving to try to address some open questions in the transition towards a fully CAV fleet on the road networks. Some of the latest studies are also introduced to figure out why simulation modelling can be a suitable tool for evaluating the CAV potential on roundabouts from an efficiency perspective.

### 2.1. The Transition towards Connected and Automated Vehicles

Connectivity and automation are emerging technologies applied in combination to increase road safety, traffic and energy efficiency, driving comfort and existing roads’ throughput [13,14]. The recent literature suggests several taxonomies on the human–automation interaction that are useful for understanding the liability concerns of the automotive manufacturers and lack-of-trust concerns of the car owners towards the replacing of the human perception and decision making with automation [9,26,27,28,29]. The Society of Automotive Engineers [9] has proposed six levels of driving automation from no driving automation (level 0), driver assistance (level 1), partial driving automation (level 2), conditional driving automation (level 3), high driving automation (level 4) to full driving automation without human participation (level 5). In turn, there is not a standardized conceptualization for the vehicle connectivity levels which are currently distinguished by different functionalities: vehicle-to-vehicle (V2V), vehicle-to-infrastructure (V2I), vehicle-to-pedestrian (V2P) and vehicle-to-network (V2N) [12,30]. 

The literature informs that CAVs operating with a cooperative adaptive cruise control system share their own data with other CAVs in the network by V2V communications, which should preferably accept short-range wireless technologies as the number of CAVs increases. So far, various advanced communication protocols have been proposed for V2V communications, such as 5G [31]. Thus, the combination of the levels of connectivity and automation plays an ever more important role in defining the contribution of the CAV technologies to the creation of the long-term benefits at the single vehicle level and at the transport system level [12,31]. It should be noted that that the net energy savings at vehicle level, however depending on the vehicle features, the powertrain design or the vehicle operations, are about 10% to 23% with fully automated and connected vehicles, but without including energy demand of the automation and connection components [12]. Although higher energy savings can be expected by a combination of high-level connectivity and automation, how automation and connectivity technologies can help to increase (or not) the energy efficiency at the transport system level is still an open research question [12,32]. There may be, however, an increased travel demand and vehicle miles travelled by individual motorized vehicles rather than the desired and increased use of the shared mobility solutions with CAVs, with the risk of an overall increase in road congestion and energy-related CO_2_ emissions [33,34]. On the other hand, there will be social challenges mainly related to the concerns on the regulatory requirements and users’ lack of trust in the progressive transition towards fully cooperative driving [34]. Another open question, indeed, concerns the level of authority assigned to the soft automation technologies (e.g., cruise control, adaptive cruise control, automated steering, collision warning system, parking aids) which drivers can override if they do not need them or if they do not want to employ them, or the hard automation technologies (e.g., automatic transmission, anti-lock braking system, traction control, electronic stability control, collision avoidance system) which have the ultimate authority over the vehicles’ actions [35]. There is still a need for in-depth human factors research to better understand the process of transferring the driving responsibility, especially where the automated or connected responses fail due to technical malfunction, and drivers must resume control employing manual driving features, where possible [36,37]. Still, in the absence of the digital connectivity, the information available to automated driving systems is limited to data gathered by on-board sensors, typically constrained by the sensor’s line of sight and the rate at which the sensors take measurements [2]. Thus, the ordinary adaptive cruise control systems provide minimum time gaps comparable to those of HDVs, thus decreasing the roadway capacity especially when their use is widespread [2,38]. In turn, the digital connectivity between vehicles, between vehicles and transport infrastructure, and between vehicles and other road users, provides the automated driving systems with more information about the surrounding environment, and it also enables the cooperative element to enhance the detection capabilities of vehicles and the adaptation of drivers’ behaviour while driving [3,23]. Compared to the conventional cruise control or adaptive speed control, the cooperative adaptive cruise control technology that is enabled by vehicle-to-vehicle communication, may safely permit a vehicle to travel more closely to a lead vehicle and may allow more vehicles to enter a lane, so as to reduce traffic jams and increase the overall average speed [2,23]. 

**Figure 2:**
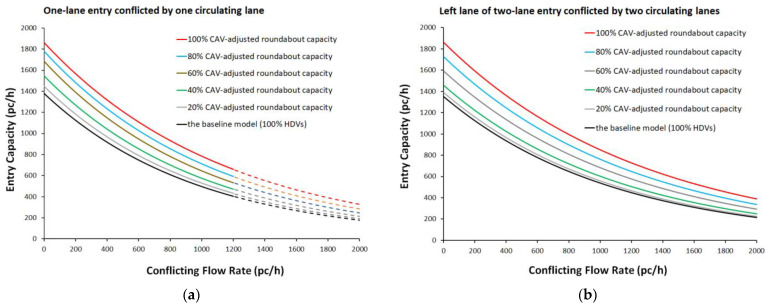
The baseline model for 100% HDVs and the CAV-adjusted roundabout capacity curves under different penetration rates of CAVs for: (**a**) the one-lane entry conflicted by one circulating lane (where dashed regression is extrapolated beyond the data); (**b**) the left entry lane of a two-lane entry conflicted by two circulating lanes (where the operational range depends on the geometry under study). Source: Own research based on data presented in [2].

Despite the special interest of the cooperative adaptive cruise control technology in the car-following and lane-changing maneuvers, the motivating forces behind them can be difficult to ascertain and may cause human error [23,39]. At present, the level 2 is the most common level of automation of vehicles (e.g., [9]) where the adaptive braking and acceleration enable the vehicle to adjust its speed without driver assistance [38]; there are not yet in production for consumers vehicles with the driving automation features of levels 4 and 5 (e.g., [9]), where the driver will be not required to take over driving when automated features are engaged [9,12]. There is also the need to fill the lack of novel models and methods to assess the presence of CAVs in traffic in the transition period towards a fully driverless option. Since CAV regulation and technology are still in development, driving simulation modelling can be conducted using CAV logic but any prediction of future traffic conditions may be used to assess the potential scenarios on what will happen with the widespread use of CAVs in traffic [23]. In this view, the 2022 Highway Capacity Manual (HCM) [2] presented the capacity adjustment factors for different penetration rates of CAVs to modify the core methodology inputs and to take into account the presence of CAVs for specific road entities [2,23]. These factors were derived from microsimulation under the assumption that all communication elements were working with a high degree of reliability; to date they are the only methodological support that responds to the transportation agencies’ need to assess the potential ability of CAVs and their long-range effects on roadways’ throughput. Figure 2 shows, by way of examples, the CAV-adjusted entry capacity relationships for roundabouts from the 2022 HCM [2]: (a) the baseline model represents a traffic stream consisting of 100% human-driven cars; (b) the CAV-adjusted roundabout capacity curves were obtained for a percentage of CAVs varying from 20% to 100% in 20% increments [2]. Thus, the reader is able to assess, for a given market penetration rate of CAVs, what percent increase of the roundabout capacity can be expected for applications.

### 2.2. Consumer Attitudes toward the Acceptability of Automated Driving Systems

Despite an increasingly positive attitude of consumers to accept automated driving systems, the transition towards new mobility solutions and business models of transport services is not free from questions about their impact on individuals and society due to a not negligible level of reluctance of the users to endorse a fully automated road network [40]. Predominant barriers to the potential of autonomous driving are mainly related to multi-faceted issues incorporating the control of transport choices and reliability, road safety and personal security, accountability in the event of a crash, and costs [31,41]. In this regard, the 2013 U.S. automotive emerging technologies study [15] investigated 16,758 car owners on their intentions to spend on autonomous driving technology. The survey showed a great interest in semi-autonomous driving features such as emergency braking and steering (40%), automatic park assists (32%), speed limit assist (20%), traffic jam assist (26%), but less interest in a fully autonomous mode (about 21% of drivers). Interest in autonomous driving features by gender showed comparable percentages especially for low-speed collision avoidance systems at market price (58% of men and 51% of women), emergency braking and steering systems (42% of men and 35% of women), autonomous driving mode (23% of men and 19% of women) and automatic park assist systems (29% of men and 32% of women). Despite the perceived benefits on road safety, personal security or saving fuel, consumers preferred the responsibility of driving their own vehicles until these technologies would have fully gained their confidence and trust. The survey on autonomous and self-driving vehicles conducted in 2014 included 3255 participants from China, India, Japan, the U.S., the U.K. and Australia [42]. Although 70% of respondents had already heard about these vehicles, only Chinese respondents were five times as likely to report a very positive opinion about the autonomous driving technologies (49.8%) than Japanese respondents (10.1%). An online survey conducted in 2015 addressed to 5000 respondents from 109 countries also investigated their purchase intentions with reference to the levels of autonomous driving [9,16]. The results showed that drivers travelling more kilometers were willing to pay more for an autonomous vehicle, whereas users spending more time driving were more willing to purchase a new autonomous car.

A 2017 survey on a sample of over 1000 participants aged between 20 and 70 years, from Italy and other European countries or the Middle East investigated the willingness of road users in using smart transport solutions in one’s city of origin [43]. The participants’ responses in the questions were highly positive about real-time information through digital interfaces and the role of public transport systems, though in a different way with regard to needs of work, family status and age. Most participants were generally positive about electronic ticketing and on-board navigation services (60%) and car sharing (40%). In turn, the respondents aged between 30 and 40 thought themselves users of on-demand public transport systems, drivers needing e-parking, users of car-sharing or bike-sharing solutions in the near future, while the horizon was shifted a bit forward for thinking themselves as future passengers of driverless cars. The 2021 experiment carried out to explore car users’ preferences towards shared autonomous mobility options also showed that the experience influenced the modal choice preferences [44]. However, new mobility services associated with connected and automated driving could increase the motorized individual transport, or the travel demand by users that are not able to drive or prefer to be driven instead of driving themselves [12]. Although innovations in vehicles are continuing to foster the transport transition towards connected and electric vehicle technologies, a person-centered taxonomy should be used in the preliminary studies on transport demand to understand what behavioural change is desirable or expected with regard to new intelligent mobility solutions [43]. According to the 2017 investigation of expectations of large-scale users [43], this kind of taxonomy can be a dynamic tool for smart transport designers and policy makers both to formulate marketing assumptions, and to better understand the recipients of proposals related to smart mobility. The 2020 survey on consumer attitudes towards connected and electric vehicles have also indicated the potential for environmental-friendly transport, travel accessibility for non-drivers and less driving fatigue as the most attractive aspects [45]. Despite the perceived benefits and infrastructure improvements, however, costs, vehicle safety and legal liability, and some technical aspects related to battery service life and charging, still represent the prominent barriers to the adoption of CAVs. Benleulmi and Ramdani [46] examined the effect of instrumental, symbolic and affective motives on the users’ intention to use fully autonomous vehicles. Based on a survey of 240 U.S. residents, the results suggested that the behavioural intention to use fully autonomous vehicles depended on fulfilling instrumental motives (e.g., performance expectancy or hedonic motivation), symbolic motives (e.g., personal innovativeness or social influence) and affective motives (e.g., trust and performance risk). These results may have implications for designing policy interventions to increase the deployment of autonomous vehicles. Another study [47] investigated the impacts of several factors on the general acceptance of autonomous vehicles including the perceived motion sickness in an autonomous vehicle, the willingness to use time more efficiently in an autonomous vehicle, the perceived value of time and the perceived risk using private SAE level 5 autonomous vehicles [9], as well as the interrelationships between these factors. Based on a total of 1418 valid surveys, the authors observed that the perceived risk and the willingness to use time more efficiently in an autonomous vehicle greatly affected the behavioural intention. The 2022 investigation on the main contributors to travelling more by autonomous vehicles analysed the data from 359 respondents who had ridden in an SAE level 3 car as a driver or a passenger [9,48]. The questionnaire queried the respondents’ user experience with the automated driving function, the barriers of travelling by car, and previous experience with advanced driving assistance systems. The study found that conditionally automated cars have a substantial potential to increase travelling by car once they will become available. However, the growth of traffic with private autonomous vehicles should be limited by the use of alternative, shared and more sustainable travel modes.

### 2.3. Simulation Modelling of CAVs on Roundabouts

The technical and scientific literature reports that autonomous driving with cooperative decision making has the potential to improve gap usage on roundabouts [8]. In a transient state there is also the need to study the key road operational issues when HDVs and CAVs are mixed in the same road or intersection at the same time. However, the question arises as to what the prospects about the performance of CAVs on roundabouts are where the curvilinear trajectories may complicate the interpretation of the intentions of the other vehicles [1,49,50]. 

Since the introduction of connected driving requires communication between vehicles, and between the vehicle and road infrastructure, there is also the further question on CAV ability to receive information from the roadway infrastructure, other road users or vehicles, service providers and traffic control centers, thus having better anticipation and sensing of the preceding vehicles’ actions. Bearing in mind that the true level 5 self-driving car should head to a roundabout as a human driver would do, a control system for a full dynamic navigation and aware-situation connected driving should have a complete realization that it is about to negotiate a roundabout where a flow circulating around the central island is established to change direction, and path planning includes entrances, turning maneuvers, merging, lane changing and exits [11,50,51]. In this regard, Pérez et al. [11] tested a control lateral system in a 3D simulator to emulate a driverless vehicle in a roundabout. The recognition of turning maneuvers and lane change trajectories defected when the vehicle was turning on the smallest radii because the steering wheel was close to its maximum capacity; thus, further studies will be required to optimise entry radii and/or the deviation from the reference circle. Martin-Gasulla and Elefteriadou [49] proposed a novel rule-based algorithm to simulate CAVs negotiating a single-lane roundabout as a central controller through V2I communications. Although the authors tested various combinations of geometry and traffic patterns to assess the operational performance, they argued that more variability in the CAV behaviour and in the traffic demand should be implemented to gain generalizable conclusions. The above considerations reinforce the importance of designing traffic management systems useful to transportation agencies to account for CAVs’ potential ability to increase the existing roadways’ capacity. 

Microscopic traffic simulation is a valuable tool to investigate the performances of design or traffic management alternatives, and to assist decision makers in the choice of the most suitable option [2]. Microscopic traffic simulation deals with individual vehicles and their interactions based on car-following, lane-changing, and gap-acceptance models [24]. There is the need to search for the values of the model parameters that will produce a valid representation of the system under study, through an iterative process that fine-tunes the model parameters, compares the model to the actual system and uses the insight gained to improve the model accuracy until the agreement with the target values is deemed acceptable [22,23]. For some time now, there have been several commercial and open-source platforms with inbuilt models or models with externalities [20]; however, they have been mainly focused on automated vehicle features (e.g., [52,53,54]). CAV simulation is now of great interest to evaluate the effects of the progressive introduction of CAVs in traffic and the specific consequences on intersection management in smart scenarios (e.g., [55,56,57]). However, the concluding remarks of the latest research have provided decision makers and practitioners with knowledge on the potential impacts of CAVs on freeway safety or capacity (e.g., [58,59]) and energy savings over human-driven vehicles (e.g., [60,61]), but relatively few studies have been done to investigate the effect of CAVs operation especially on roundabouts. And still, few dynamic cooperative models for connected and automated vehicles were derived from the actual responses measured in the field or applied to simulate multi-vehicle car-following scenarios (e.g., [62]). Despite being at an experimental stage, there are lots of questions about the use of microscopic traffic simulation models for CAVs on roundabouts. Anagnostopoulos and Kehagia [51] used VISSIM software [63] and the Surrogate Safety Assessment Model (SSAM) [64] to analyse the total conflicts with CAVs on double-lane roundabouts. Despite significant increases in road safety under high penetration rates of CAVs in traffic, there were slightly increases in total conflicts in cases where shorter gaps were accepted; in turn, there were increases in the emissions of carbon dioxide and nitrogen oxides where CAVs behaved with a defensive driving style. Virdi et al. [17] performed a comparative safety assessment for priority-controlled intersections under mixed fleets using the SSAM [64]; the analysis returned greater reductions of potential conflicts on multi-lane roundabouts and give-way intersections than signalised intersections. Giuffrè et al. [65] showed safety improvements in the progressive transition to full automation on turbo-roundabouts particularly in presence of internal traffic separations in the turbo-block. 

Microsimulation-based modelling has been also employed to investigate the potential benefits of the automated driving systems on roundabout efficiency (e.g., [66,67]). The main conclusions concerned the beneficial effect on traffic as the penetration rates increased, whereas low percentages reduced efficiency especially where dedicated lanes were introduced. In this regard, the potential of the cooperative driving in negotiating single-lane roundabouts has been investigated to assess improvements in capacity and delay time [50]; the results showed that high penetration rates of assertive CAVs returned increases in capacity by 58% to 73%, while the average control delay reduced from 80% to 97% compared to traffic with human-driven vehicles. Despite insights for more efficient traffic management, maximizing throughput could be really obtained thanks to a better coordination between vehicles [68]. The role of geometry and its effects on the expected performances should be assessed with reference to the driving styles that CAVs may actually perform in the progressive transition towards a fully connected driving. A two-stage optimization model to optimise vehicle trajectories through a single-lane roundabout has been proposed by [69] to improve the operational performance under the fully CAV environment. The authors started from considering explicitly the geometric features of roundabouts in the field and performed throughput and delay comparisons. Although the findings of this research offered new insights into the mechanism of controlling CAVs on single-lane roundabouts, the authors argued that further layouts of roundabouts should be examined also for different penetration rates of CAVs. However, there is still a great coexistence of HDVs and CAVs that should be better understood before the advent of the fully CAV environment. Thus, although many models and algorithms have been proposed for intersection control in the CAV environment, the amount of research on roundabouts is still limited. In this view, the paper is a contribution towards bridging this gap by analysing the impact of CAVs on roundabouts from an operational perspective. The versatility of Aimsun Next [25] has been also tested to evaluate the possible effects of the progressive introduction of CAVs in traffic and the specific consequences on the efficiency of the examined roundabout layouts.

## 3. Materials and Methods

This section describes the steps we have taken in Aimsun Next [25] to model the presence of autonomous vehicles equipped with the cooperative adaptive cruise control on roundabouts, and to assess their potential impact compared to the baseline scenario with 100% human-driven vehicles. Operations at capacity were simulated since CAVs in traffic are expected to create an incentive to operate the entry mechanisms at a high level of utilization. Specifically, utilization can be thought of as the ratio of the number of entering vehicles (i.e., the throughput) to the maximum number of vehicles that each entry lane could serve (i.e., the capacity). Capacity calculations were based on the capacity models and the adjustment factors for CAVs proposed by the 2022 HCM for roundabouts [2]. This section also presents the assumptions behind the fine-tuning process of the model parameters that we made, while the results about the “what-if” scenarios analysis which we performed in Aimsun Next [25] will be presented in Section 4.

### 3.1. Setting Up Roundabouts in the Aimsun Next Environment

**Figure 3:**
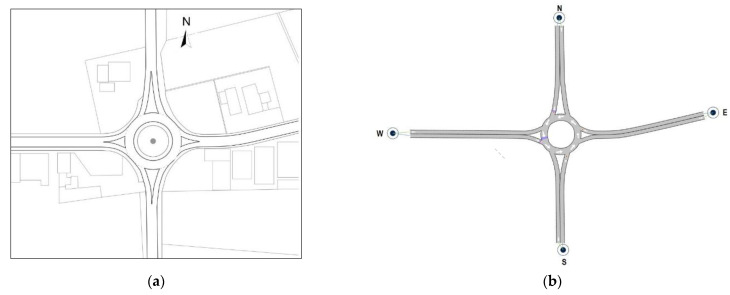
The single-lane roundabout (roundabout 1): (**a**) the sketch of the roundabout; (**b**) the network model built in Aimsun Next with labels for the centroids (N: North; S: South; E: East; W: West).

**Figure 4:**
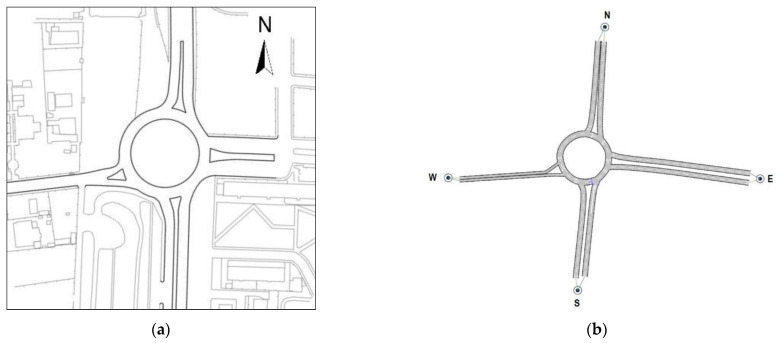
The two-lane roundabout (roundabout 2): (**a**) the sketch of the roundabout; (**b**) the network model built in Aimsun Next with labels for the centroids (N: North; S: South; E: East; W: West).

In order to examine the mechanisms of entry introduced in Section 1, two existing roundabouts were identified: a single-lane roundabout with a one-lane entry conflicted by one circulating lane was the reference for examining the entry mechanism 1 (see Figure 3a); a two-lane roundabout where the left lane of the two-lane entry is conflicted by two circulating lanes was the reference for examining the entry mechanism 2 (see Figure 4a). Thus, the roundabouts’ network models which were then built in Aimsun Next (see Figure 3b and Figure 4b) were based on the geometry and traffic flows measured in the field to reflect the functional scope of actual roundabout systems. It should be noted that the 2022 HCM [2] has proposed two different roundabout capacity models for each lane of a two-lane entry conflicted by two circulating lanes. In this study only the left entry lane was considered to examine the entry mechanism 2, since surveys in the field on the two-lane roundabout in Figure 4a highlighted that the vehicles entering from the right entry lane mainly exited just past the entrance, so that, de facto, a mechanism of entry with one antagonist traffic stream occurred at the right entry lane.

The single-lane roundabout in Figure 3a is located in western Sicily, Italy, at the intersection of Salemi Street heading eastbound toward the City of Mazara del Vallo, the SP 50 provincial highway westwards, and the highway 115 in the direction south–north and north–south, 400 m from the route E90; the west entry lane in Figure 3a was the subject approach for exploring the entry mechanism 1 with a one-lane entry conflicted by one circulating lane. The geometry of the single-lane roundabout in Figure 3a included a 39 m outer diameter between the outer edges of the ring roadway, a 7.00 m wide circulatory lane, a 4.50 m wide single lane on entries and exits on the major roads in the north–south direction, and a 4.00 m wide single lane on entries and exits on the minor roads identified in the east–west direction; the roundabout design also included a non-traversable central island, raised splitter islands and deflection angles greater than 45 degrees (see e.g., [70]). Field surveys highlighted a mix of large warehouses and small-scale non-residential buildings causing a low likelihood of pedestrian activity; a high frequency of the private mode compared to the public counterpart is also due to a ring road as an alternative route to heavy traffic from the Port of Mazara del Vallo to the motorway. Traffic volumes by entry lane were recorded during morning peak (from 8:00 am to 9:00 am) and afternoon peak (from 7:00 pm to 8:00 pm) in March and April 2022. The extracted data returned the mean value of entry traffic flows equal to 1310 vehicles per hour, then used as input to set up the roundabout in Aimsun Next. Surveys revealed a traffic consisting of 84% of cars, 2% of motorcycles, 8% of vans, 1% of bikes and 5% of buses and trucks. In turn, Figure 4b shows the two-lane roundabout operating in the road network of Palermo City, Italy, where the left lane of the northbound entry was the reference for examining the entry mechanism 2. This suburban roundabout is sited in an urbanistic context similar to the previous intersection and is located among Lanza di Scalea Street (that is headed north to the A29 motorway and south to the city centre), Besta Street (that is directed west towards the village of San Lorenzo) and Einaudi Street (that is directed east towards the Zen neighborhood). There is a 71 m outer diameter, a 4.00 m wide entry and exit lanes and 8.00 m wide circulatory roadway. The roundabout design included a non-traversable central island and raised splitter islands; deflection angles are greater than 43 degrees for all the entry approaches. 

Traffic flow data were collected both manually and videotaped on each entry (and exit) during the morning peak (from 8:00 am to 9:00 am) and afternoon peak (from 7 pm to 8 pm) on weekdays in May 2022; a total entry flow of 3422 vehicles per hour was registered during surveys with about 11% of heavy vehicles and 5% of motorcycles, while pedestrian flows were insignificant due to the predominantly non-residential feature of the site and the absence of commercial areas. In both the roundabouts the speed limit is 50 km/h and traffic flows from all the legs were balanced; the 85th percentile speeds were consistent with the literature on suburban roundabouts [71]. Roundabout initial setting up in Aimsun Next consisted of creating the roundabout network models using the geometry collected in the field; each roundabout was modelled by building each approach road as a give-way link and the roadway around the central island as a series of prioritized-featured links. All the road sections forming the entries and exits were first defined by specifying the infrastructure type “Roundabout” and the number of lanes in the *Project folder* of Aimsun. The network models of each roundabout have been adjusted so as to ensure that the origin and destination lanes were correctly linked (see Figure 3b and Figure 4b). Detectors were placed on the sections of the network models at the desired location where their measuring capability was used to check the evolution of the traffic parameters during the simulation. 

### 3.2. The Capacity Target Values in the “What-If” Scenarios Analysis

According to the objectives of the paper, the entry capacity models proposed by [2] were applied to perform a “what-if” scenarios analysis for CAVs navigating roundabouts:

(1) $$C_{e,adj,CAVs}=f_{A}\cdot A\cdot e^{-f_{B}\cdot B\cdot Q_{c}},$$

where: *C_e,adj,CAVs_* is the CAV-adjusted entry lane capacity (pc/h) as a function of the conflicting flow rate *Q_c_* (pc/h); *A* and *B* are the parameters controlling the intercept and the slope of the capacity curve, respectively; the factors *f_A_* and *f_B_* adjust the intercept parameter *A* and the slope parameter *B*, respectively. Table 1 shows the adjustment factors for different proportions of CAVs in traffic to examine the entry mechanism 1 where the one-lane entry is conflicted by one circulating lane (see the west single-lane entry in Figure 3b), and the entry mechanism 2 where the left entry lane of the two-lane approach is conflicted by two circulating lanes (see the south entry in Figure 4b). 

In the entry mechanism 1 *A* and *B* were set equal to 1380 and 1.02 × 10^−3^, respectively [2]; in turn, the entry mechanism 2 *A* and *B* were set equal to 1350 and 0.92 × 10^−3^, respectively. All other factors being equal, the entry lane capacity will increase where *A* increases or *B* decreases. The capacity function expressed by Equation (1) returned the entry capacity target values which were used as a reference to compare the simulated data. Aimsun Next to delete typos [25] was used to reproduce operation at capacity on each roundabout network model. First, the traffic demand in the *Traffic Demand folder* of Aimsun Next was preliminarily created in the form of an origin–destination matrix (OD = O_i=1,..n_; D_j=1,..,n_; n = 4) from each origin *i* to each destination *j* to reproduce the trips between the OD centroids (see Figure 3b and Figure 4b). The time intervals to apply the traffic demand at the roundabouts 1 and 2 were set starting from the initial time at 8:00 am and 7:00 pm, respectively. To assess the capability of Aimsun Next to reproduce the observed traffic, simulation started as follows: 15 min initialization to load traffic into the network and to reach an equilibrium condition; 60 min simulation and then 10 min completion to empty the system without compromising simulation quality. The Geoffrey E. Havers (GEH) index [24] resulted smaller than 5 in more than 85% of the cases when the simulated flow rates were compared with the data detected at entries in each roundabout throughout each 5 min sampling interval in the peak hours. The total traffic matrix for each roundabout was then split into two OD matrices (i.e., a matrix for CAVs and a matrix for HDVs) based on the penetration rate of CAVs which varied by each scenario. The full range of traffic flows, from free flowing up to capacity, has been reproduced by means of subsequent OD matrices, but each entry never became congested, in the sense of experiencing stop-and-go traffic; thus, the traffic flows entering from the subject entry lane were conflicted by a circulating traffic gradually increased in steps of 200 veh/h. Concerning the entry mechanism 1, a total of 7 subsequent OD matrices have been built and assigned to each single-lane entry so that the traffic flows entering from the subject entry lane conflicted by a circulating traffic of CAVs and HDVs, gradually increased from 0 to 1200 veh/h in steps of 200 veh/h. In the case of the entry mechanism 2, a total of 9 subsequent OD matrices have been defined and assigned to all the entries so that the traffic flows entering from the subject left entry lane conflicted by two circulating traffic streams of CAVs and HDVs, gradually increased from 0 to 1600 veh/h in steps of 200 veh/h. In both cases, when a saturated condition was being reached, the maximum number of vehicles moving out to the entrance line to enter the roundabout provided the entry lane capacity [2]. Once the OD matrices were defined for both roundabouts, simulation started with the default values of the model parameters. Aimsun Next [25] was used to model the CAV driving in the transition to an all-CAV traffic fleet. Thereafter, there was the need to improve the degree of closeness between the capacity target values calculated using Equation (1) and the simulated data by fine-tuning the model parameters. The next section presents the results of the process that allowed us to explore the effects of the variations in the values of the model parameters on the gap-acceptance behaviour at entries under heterogeneous traffic.

### 3.3. Fine-Tuning the Model Parameters with Effects on CAVs

Modelling enhancements include the following: the ability to adjust behavioural parameters to characterise a vehicle implementing decision-making processes and behaving more or less cautiously where it moves to change lane or to accept a gap, maintaining a longer or shorter headway from the preceding vehicle, turning lane with more or less anticipation, or being prudent or aggressive in lane changing, and so on. 

The CAV models of Aimsun Next [25] are based on experimental data both on vehicles equipped with the cooperative adaptive cruise control or the adaptive cruise control only [59]. An initial sensitivity analysis was performed to identify the values for the model parameters that affect the entry mechanisms under CAV-based assumptions and cause the model to best reproduce the capacity target values. The idea behind the fine-tuning process of the model parameters is that the capacity functions calculated using Equation (1) and the capacity adjustment factors in Table 1, were the capacity target curves to be used to compare the simulation output. According to [24], the 2022 HCM [2] was used as a source of target values of entry capacity since the field measurements of traffic flows were infeasible due to the current lack of significant proportions of CAVs in traffic; this is also due to existing road infrastructures that are not yet smart enough to fully support the cooperative driving. Based on a specific level of agreement between the CAV-adjusted and simulated capacities under different market penetration rates of CAVs in traffic, the simulation model could then be accepted since it replicated as closely and accurately as possible the phenomenon under analysis [24]. 

Aimsun Next was executed to investigate the following “what-if” traffic scenarios: the baseline scenario consisting of 0% CAVs and 100% HDVs; scenario 1 consisting of 20% CAVs and 80% HDVs; scenario 2 consisting of 40% CAVs and 60% HDVs; scenario 3 consisting of 60% CAVs and 40% HDVs; scenario 4 consisting of 80% CAVs and 20% HDVs; and scenario 5 consisting of 100% CAVs and 0% HDVs. The curve corresponding to the baseline scenario consisting of 100% HDVs and 0% CAVs was calculated using Equation (1) with *f_A_* and *f_B_* = 1.00 (see Table 1); the same equation was used to calculate the target curves in each scenario where the capacity adjustment factors *f_A_* and *f_B_* varied as shown in Table 1. It is well known that the car-following, lane-changing, and gap-acceptance rules govern the interactions between individual vehicles within Aimsun Next [25] as for other microscopic traffic simulation models [24]. The vehicle longitudinal behaviour is based on the leader vehicle according to the car-following model, while the vehicle lateral movement when the vehicle is changing lane is described according to the lane-changing model [24]. The presence of CAVs in traffic specifies the gap-acceptance process at the entrance: a CAV negotiating a roundabout activates the cooperative adaptive cruise control system whether the conflicting vehicle on the circulatory roadway is also a CAV to acquire information on the location or speed of the conflicting CAV and to accept or reject the gap; in turn, a CAV at the yield line activates the adaptive cruise control only to enter the roundabout whether the conflicting vehicle is a HDV [2]. There is in addition a lane-changing chance on the two-lane circulatory roadway so that the differences between the CAVs and HDVs in the driving behaviour and yielding process at entries get more complicated than the single-lane counterpart. A sensitivity analysis and manual fine-tuning were made to identify the values of the parameters that caused the model to best reproduce the capacity target values. The literature about the fine-tuning process of the model parameters recommends using the fewest number of model parameters as possible, dealing with them individually by a sensitivity analysis and adjusting them in the microscopic simulator depending on the effect on the simulation output, then running the simulation several times to adjust the model parameters iteratively and to return outputs as close as possible to the target values of the considered variables [24,72]. Table 2 depicts the various vehicle parameters of Aimsun Next [25] used for the fine-tuning purposes as introduced above.

The values of the model parameters for the base-line scenario were calibrated in previous studies by the authors for single-lane and two-lane roundabouts where human-driven cars only in line with the Italian vehicle types were in traffic [73,74]. The set of the parameters in the *Dynamic Models tab* of Aimsun Next [25] for the baseline scenario included (see also [24]):

- The driver reaction time or the time it takes a driver to react to speed changes in the preceding vehicle; lower reaction time means higher capacity, so that the vehicles can drive closer to the preceding vehicles and find gaps more easily to enter the network. Higher capacity occurs with lower reaction time where the vehicle can drive closer to the preceding vehicle, accepts and finds gaps more easily to enter the network;
- The speed limit acceptance that can be interpreted as the “level of goodness” of the drivers or the degree of acceptance of the speed limit: when it is greater than 1 means that the vehicle will take as maximum speed on a given section a value greater than the speed limit, while when it is lower than 1 means that the vehicle will use a lower speed limit;
- The gap or the time between the rear bumper of a vehicle and the front bumper of the following vehicle. This parameter can be fine-tuned to override the headway between the vehicles: the default value of 0.00 s implies that the headway between the vehicles, measured from the front bumper to the front bumper, will be used, while any other value will force a larger headway. Since the deceleration component of the car-following model is affected by the constraints imposed by the preceding vehicle when it tries to reach the desired speed, this parameter can limit the deceleration component before updating the position and the speed of the leader vehicle respect to the follower vehicle.

It should be noted that the reaction time is a car-following parameter of Aimsun Next that is usually set constant during simulation for the vehicles of a single user class (e.g., HDVs or CAVs); it is equal to the simulation time step so that each driver reacts to the speed variations in the preceding vehicle immediately at the next time step. Since the HDVs have higher reaction times than CAVs, there was the need to calculate a weighted average of the reaction times that were fine-tuned for both user classes; the weights were assumed equal to the proportions of each user class (i.e., CAVs or HDVs) in each “what-if” scenario. The sensitivity analysis also included further parameters as the clearance (i.e., the distance in meters that a vehicle keeps between itself and the preceding vehicle when stopped) and the lateral clearance (i.e., the minimum lateral spacing between two vehicles or the sum of the lateral clearances of both vehicles); however, they turned out to be not influential concerning the longitudinal and lateral behaviour. The length and width of vehicles were assumed equal for both vehicle classes implemented in the testing simulations where the driving behaviour, however, was differentiated based on the type of the leading vehicle (i.e., a shorter gap occurs only if a CAV follows another CAV). The set of the modelling parameters for the traffic scenarios with CAVs, in turn, included:

- The maximum acceleration that measures the attainable maximum value by a vehicle in any circumstance; according to [75], a higher value than the default one provides better vehicular performance;
- The safety margin factor that determines when a vehicle can move at a priority junction: a higher value than the default one means more cautious driving behaviour (i.e., larger headway), otherwise, more assertive driving is expected. The adjusted value of the safety margin employed here is consistent with the recommendations by Aimsun Next where this parameter can be adjusted for a specific maneuvre to reflect the road geometry under examination [25];
- In the deceleration component of the car-following model, the sensitivity factor enables the follower to estimate the deceleration of the leader. Aimsun Next [25] allows the analyst to adjust the vehicle headway distribution to reflect cautious driving (i.e., larger headways), otherwise assertive driving (i.e., shorter headways). Thus, a value of the sensitivity factor greater than 1.00 reflects cautious driving, while a value of the sensitivity factor below 1.00 reflects assertive driving (see Table 2). The value that we chose expressed a trade-off to simulate the changes in driving behaviour or the interactions among different vehicles to evaluate the CAV driving skills in mixed traffic conditions.

**Figure 5:**
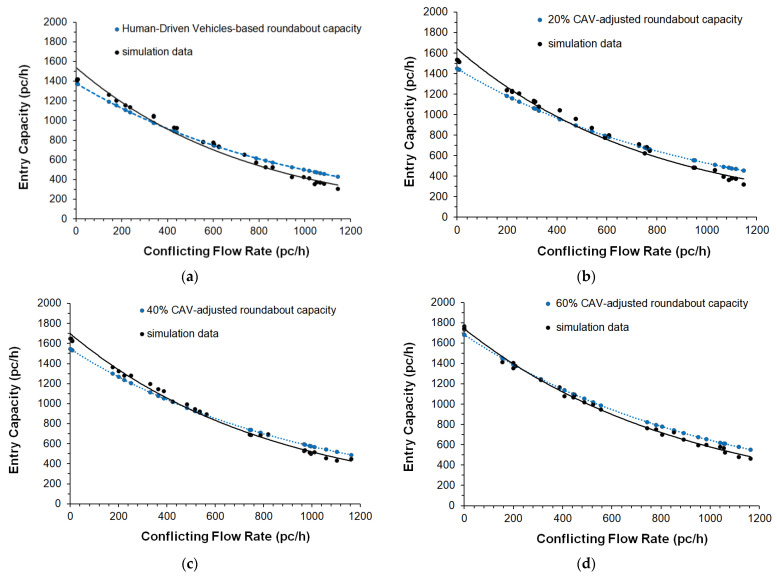

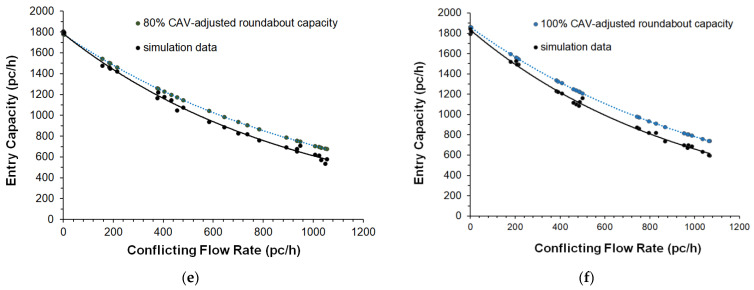
Comparisons between the CAV-adjusted and simulated entry capacity data for different market penetration rates of CAVs and HDVs for the entry mechanism 1 (i.e., one-lane entry conflicted by one circulating lane) listed as: (**a**) baseline scenario: 0% CAVs and 100% HDVs; (**b**) scenario 1: 20% CAVs and 80% HDVs; (**c**) scenario 2: 40% CAVs and 60% HVs; (**d**) scenario 3: 60% CAVs and 40% HDVs; (**e**) scenario 4: 80% CAVs and 20% HDVs; (**f**) scenario 5: 100% CAVs and 0% HDVs. Notes: to explain the entry mechanisms 1 reference has been made to the eastbound entry in Figure 3a; the black line and dotted blue line regressions were extrapolated by the data.

**Figure 6:**
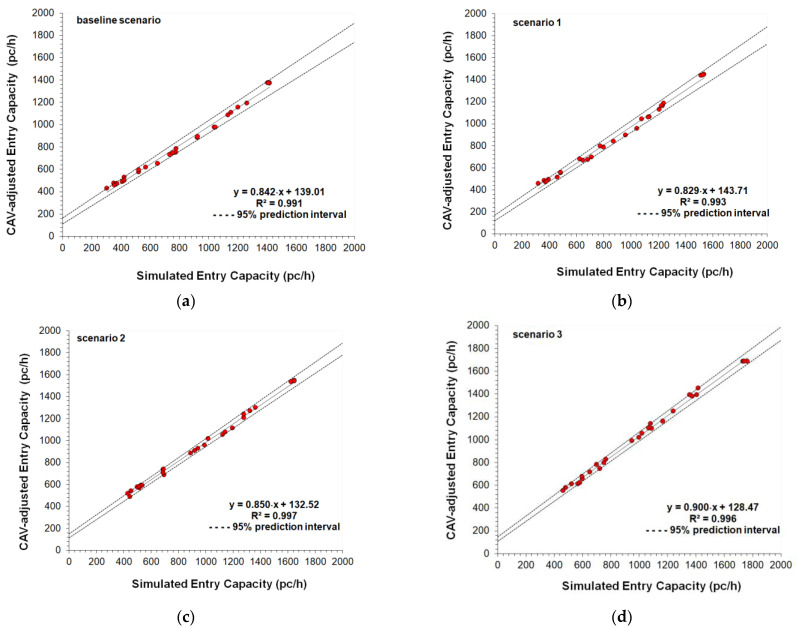

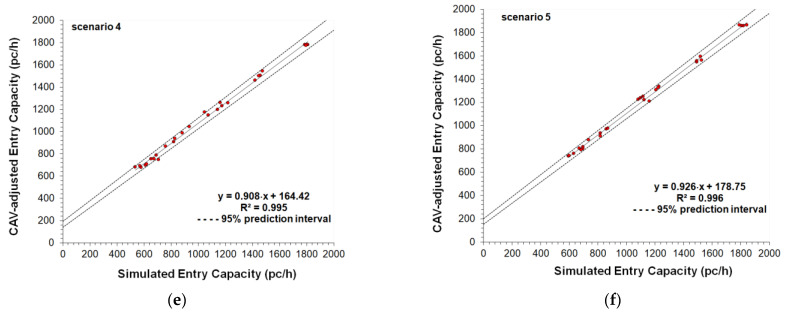
Scattergram analysis to compare the CAV-adjusted and simulated entry capacity data for different proportion of CAVs and HDVs for the entry mechanism 1 (i.e., one-lane entry conflicted by one circulating lane) listed as: (**a**) baseline scenario: 0% CAVs and 100% HDVs; (**b**) scenario 1: 20% CAVs and 80% HDVs; (**c**) scenario 2: 40% CAVs and 60% HVs; (**d**) scenario 3: 60% CAVs and 40% HDVs; (**e**) scenario 4: 80% CAVs and 20% HDVs; (**f**) scenario 5: 100% CAVs and 0% HDVs. Note: to explain the entry mechanisms 1 reference has been made to the eastbound entry in Figure 3a.

**Figure 7:**
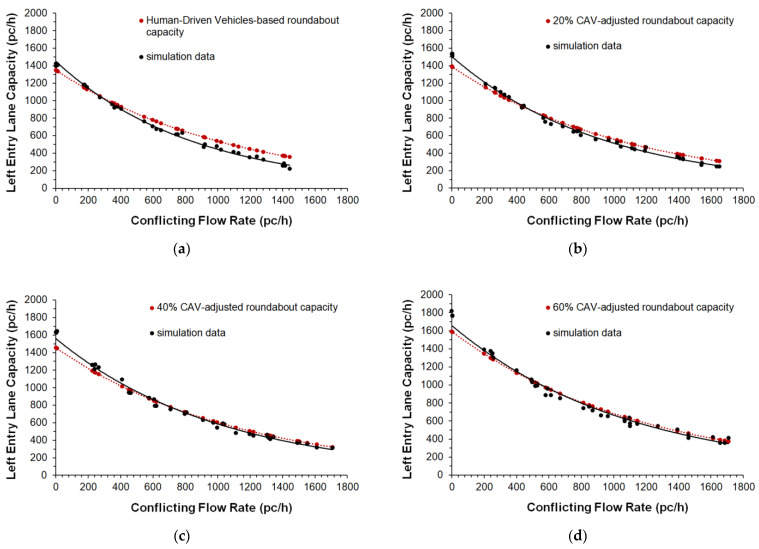

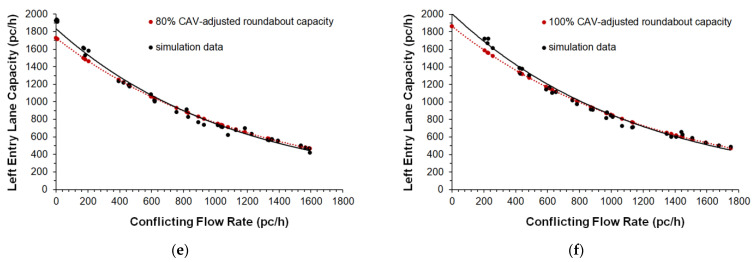
Comparisons between the CAV-adjusted and simulated entry capacity data for different market penetration rates of CAVs and HDVs for the entry mechanism 2 (i.e., the left entry lane of two-lane entry approach conflicted by two circulating lanes) listed as: (**a**) baseline scenario: 0% CAVs and 100% HDVs; (**b**) scenario 1: 20% CAVs and 80% HDVs; (**c**) scenario 2: 40% CAVs and 60% HVs; (**d**) scenario 3: 60% CAVs and 40% HDVs; (**e**) scenario 4: 80% CAVs and 20% HDVs; (**f**) scenario 5: 100% CAVs and 0% HDVs. Notes: to explain the entry mechanisms 2 reference has been made to the northbound left entry lane entry in Figure 4a; the black line and dotted red line regressions were extrapolated by the data.

**Figure 8:**
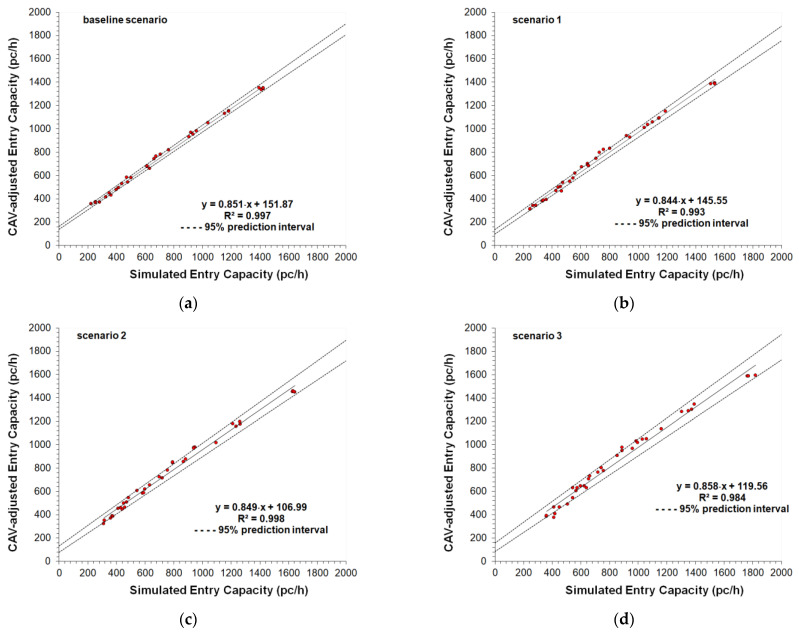

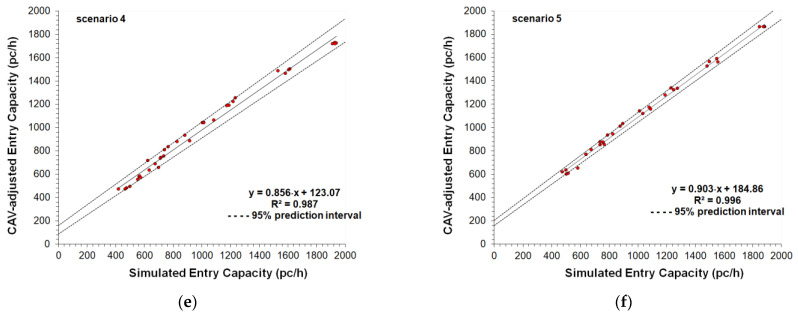
Scattergram analysis to compare the CAV-adjusted versus simulated entry capacity data for different proportion of CAVs and HDVs for the entry mechanism 2 (i.e., the left entry lane of two-lane entry approach conflicted by two circulating lanes) listed as: (**a**) baseline scenario: 0% CAVs and 100% HDVs; (**b**) scenario 1: 20% CAVs and 80% HDVs; (**c**) scenario 2: 40% CAVs and 60% HDVs; (**d**) scenario 3: 60% CAVs and 40% HDVs; (**e**) scenario 4: 80% CAVs and 20% HDVs; (**f**) scenario 5: 100% CAVs and 0% HDVs. Note: to explain the entry mechanisms 2 reference has been made to the northbound left entry lane entry in Figure 4a.

The parameter called cooperate in creating a gap was also activated in the *Cruise Control Status* of Aimsun Next [25]; specifically, this parameter ranging from 0.00 to 1.00 (where the value of 1.00 means high aggressiveness) can affect the entry mechanism 2, since at the two-lane roundabout the vehicles can cooperate in creating a gap to be accepted for a lane change. A moderate aggressiveness level of 0.50 was set also taking into account the speed limit on the roundabouts. Other model parameters such as the normal deceleration (i.e., the maximum deceleration that the vehicle can use under normal conditions), the maximum deceleration (i.e., the most severe braking that a vehicle can apply under special circumstances, such as emergency braking in front of a traffic light), the headway aggressiveness that modifies the relationship of the inter-vehicle distance as a function of the speed, were also tested, however, without the expected benefit on the fine-tuning of the model parameters. At last, the fine-tuned values of the parameters in Table 2 express a realistic trade-off in order to avoid large enough headways to significantly reduce the entry capacity, or short enough headways to return excessively improbable increases in capacity. The comparisons of the capacity target values with the simulated capacities, and the scattergram analysis for the entry mechanism 1 are shown in Figure 5 and Figure 6, respectively; Figure 7 and Figure 8 show the analogous comparisons and the scattergram analysis for the entry mechanism 2. Specifically, Figure 5 and Figure 7 show the comparisons between the CAV-adjusted and simulated capacities under different CAV penetration rates in traffic for the entry mechanism 1 and the entry mechanism 2, respectively. In all the scenarios, the entry capacity is progressively reduced as the circulating flow increased; in turn, the entry capacity increased as the penetration rates of CAVs increased_._ In turn, Figure 6 and Figure 8 show the corresponding scattergram analysis for the entry mechanism 1 and the entry mechanism 2, respectively; these graphs show the regression lines of the CAV-adjusted versus the simulated capacities plotted along with the 95% prediction intervals. Thus, the models can be accepted as they replicated as accurately as possible the phenomenon under analysis based on a specific level of agreement between the CAV-adjusted and simulated entry capacities for the different simulation scenarios [24]. 

The two-sample *t*-test was applied to verify whether the average difference between the two data subsets of CAV-adjusted and simulated data for each roundabout was really significant or if it was due instead to random chance. The *t*-statistic was determined to test for the null hypothesis (*H*_0_*: μ*_1_ *= μ*_2_) that there was no significant difference between the means of the two samples or to reject the null hypothesis that the two means were equal if |*t*| > *t*-critical value of the *t* distribution with *N* degrees of freedom at the significance level α = 0.05. To test the equality of sample variances the *F*-statistic was also calculated. Just to give an example, Table 3 depicts the summary of the results for the entry mechanism 2 where the left entry lane of the two-lane entry is conflicted by two circulating traffic streams on the inner and the outer circulatory roadway.

There is not enough evidence to reject both the null hypothesis that two means were equal and the null hypothesis that the two sample variances were equal at the significance level α = 0.05. The GEH statistic in Table 3 confirmed that the simulated values were close enough to the capacity target values (i.e., the deviation of the simulated data with respect to the target values resulted smaller than 5 in more than 90% of the cases), then the model was considered “calibrated” in terms of its ability to reproduce the capacity target values in each scenario. The results of the root mean squared normalized error (RMSNE) as referred by [24], which provided information on the magnitude of the errors relative to the average measurement, confirmed the considerations above. Although not reported here for reasons of synthesis, analogous results were obtained for the entry mechanism 1 where the one entry lane is conflicted by one circulating lane.

## 4. Results

**Figure 9:**
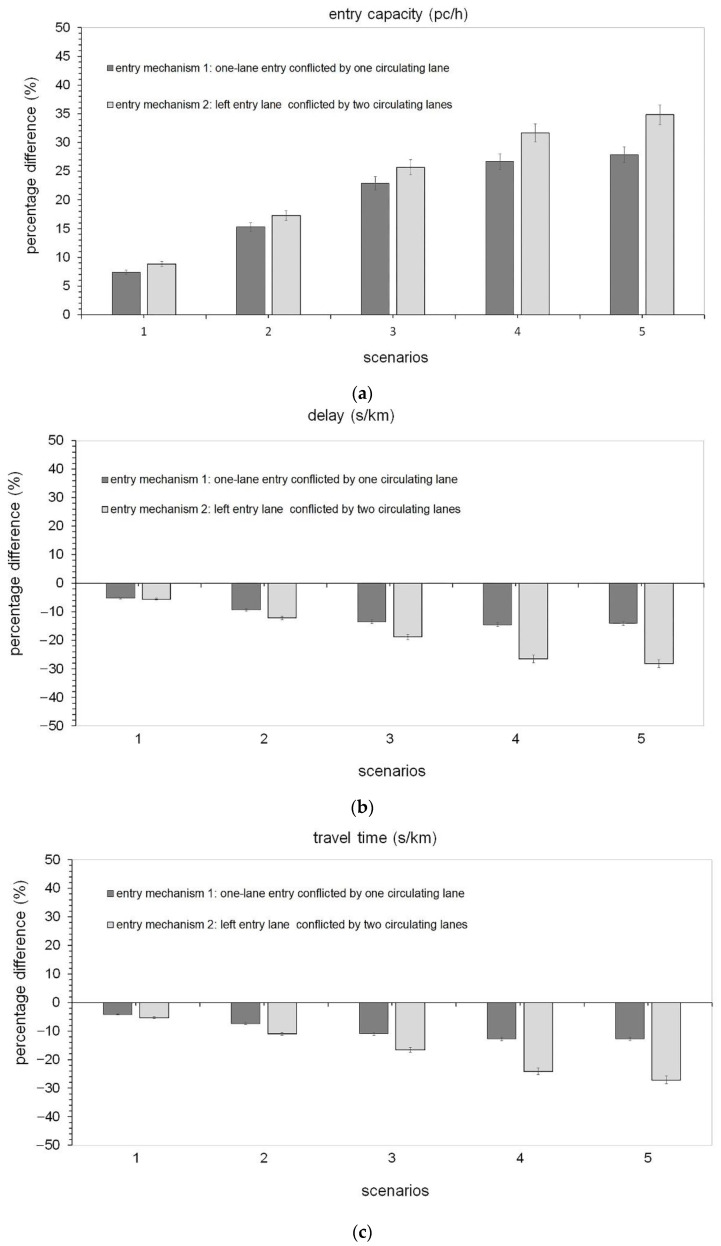
Percentage differences in the “what-if” scenarios compared to the baseline scenario with HDVs only: (**a**) entry capacity, (**b**) delay and (**c**) travel time. Note: (1) 20% CAVs vs. baseline scenario; (2) 40% CAVs vs. baseline scenario; (3) 60% CAVs vs. baseline scenario; (4) 80% CAVs vs. baseline scenario; (5) 100% CAVs vs. baseline scenario.

The analysis of the “what-if” scenarios, as the name implies, allowed us to ask the question “What if the conceptualized situation from a certain traffic scenario on roundabouts really happens?”. Microscopic traffic simulation was used to model CAVs on roundabouts and to assess the effects of their presence in traffic from an efficiency perspective. Thus, the examples of traffic scenarios designed to describe the potential associated to transitioning towards an all-CAV fleet included changes in values of the capacity, delay and travel time with reference to the mixed fleets of CAVs and HDVs that were compared to the baseline scenario (i.e., a fleet only made by human-driven vehicles). The results of the “what-if” scenarios analysis showed improved operating conditions with the increase in the percentage of CAVs in traffic. Figure 9 shows the percentage differences about (a) the entry capacity, (b) the delay and (c) the travel time compared to the baseline scenario with HDS only. These indicators were considered representative enough to support the considerations from an efficiency perspective. Specifically, the entry mechanisms considered here were used to assess the impacts of the CAVs on the examined roundabouts where operations at capacity on entries have been simulated in Aimsun Next. The entry capacity corresponded to the maximum number of vehicles moving out to the yield line at the subject entry where a saturated condition was being reached [1]. In turn, the delay time was the time loss for the vehicles compared with free-flowing traffic, while the travel time expressed the total possible routes experienced by all the vehicles as returned by the detectors on each roundabout network model [1,2,25]. The results in Figure 9a aligned with what we have derived from [67] concerning the impact of autonomous driving on roundabout capacity. The simulations showed the beneficial effect on entry capacity under steady increases in the percentage of the CAVs in each scenario (Figure 9a); among the other things, the increase in the CAV penetration rates increased the likelihood of accepting shorter gaps so that vehicles used them efficiently during the runs in Aimsun Next. To take some examples, a capacity increase of 15.00% for the entry mechanism 1 and a capacity increase of 17.00% for the entry mechanism 2 were observed in scenario 2 (with 40% of CAVs) compared to the baseline scenario with 100% of HDVs (see Figure 9a). In turn, the entry capacity increased by 23.00% for the entry mechanism 1 in scenario 3 (with 60% of CAVs) compared to the baseline scenario; the increase of 26.00% can be observed in the same scenario in Figure 9a for the left entry lane in the two-lane entry conflicted by two circulating traffic streams (i.e., the mechanism 2).

There is also a clear impact of delays and travel times on roundabout performance where the increase in penetration rates of CAVs improved the traffic efficiency. In case of the scenario 3 with the CAV penetration rate of 60%, there was a reduction of the delay and travel time values for the single-lane entry, with percentage differences up to 13.50% and 10.95%, respectively (see Figure 9b,c). There was a more significant reduction of the delay and travel time values for the left entry lane in the two-lane entry, with percentage differences of 18.78% and 16.68%, respectively (see Figure 9b,c). It should be also noted that the percentage differences of delay and travel time tend to be stabilized transitioning towards high percentages of CAVs for both entry mechanisms here considered (see Figure 9b,c). Similar trends were shown by [76], regarding the impact of situation-aware CAVs on signalized intersections. Although the progressive transition to an all-CAV fleet showed clear benefits on the efficiency of the entry mechanisms examined here, the traffic scenarios executed in Aimsun Next [25] under CAV logic should be considered as examples of what may happen based on the assumptions that we made with CAVs in traffic rather than being thought of as what will really happen with fully operational CAVs on the road network. Despite the similar impacts on traffic performance for both the two roundabouts, there were significant differences probably due to their different traffic patterns and geometric characteristics.

The “what-if” scenarios with CAVs allowed us to clarify the contribution of the geometric shape of each roundabout (in terms of size of the outer diameter, number of entry or exit lanes, and number of lanes on the circulatory roadway) on their respective efficiency; the width of 4 m characterised both entry lanes on both legs selected on the examined roundabouts as reference to explore the entry mechanisms 1 and 2. The roundabout 1 in Figure 3a was employed to study the entry mechanism 1 where the one-lane entry is conflicted by one circulating lane so that the size of the circulatory roadway and the entry (or exit) geometric design of the legs cannot admit lane changing on them. The roundabout 2 in Figure 4a was employed to examine the entry mechanism 2 where the left entry lane of a two-lane entry approach is conflicted by two circulating lanes; thus, the chance of lane changing can be admitted (i.e., two vehicles can advance side by side or, depending on the availability of acceptable gaps, lane change is possible). However, different layouts of roundabouts should be studied to better understand how the improvement of roundabout design standards could be done to update the existing road network in terms of traffic efficiency and to make it appropriate for the progressive introduction of the CAVs in traffic. Due to the worldwide spread of roundabouts, there is also the need in the medium to long-range planning to have methods to assess the CAVs’ potential capability in order to optimise driving profiles of individual vehicles and traffic flows, or to enhance their operating throughput with intelligent intersection management.

## 5. Discussion

A common consideration from the literature on connected and automated driving concerns the users’ attitudes on how quickly the CAV technologies can be adopted to improve the efficiency of the road infrastructure during use. Drivers travelling more kilometers, as revealed from user surveys, were willing to pay more for an autonomous vehicle, whereas users spending more time driving were more willing to purchase a new autonomous car [16]. Despite limited experience in road users with innovative services such as the driverless cars or dynamic queue management, the passenger-oriented awareness is likely to be key to identify highly innovative services for smart mobility with impact on future personal life of road users [43]. However, new mobility services associated with connected and automated driving could increase the motorized individual road transport, or the travel demand by people that are not able to drive or prefer to be driven instead of driving themselves [12]. There is the need of a person-centered taxonomy to be used in the preliminary studies on transport demand to understand what behavioural change is desirable or expected from implementing new intelligent mobility solutions [43]. However, field measurements are still infeasible given the absence of full levels 4 and 5 in traffic, since they are not yet in production for consumers [2]. There is also the need to fill the lack of novel models and methods to account for the presence of the CAVs in traffic during the transition period towards a fully CAV option. Since the CAV technology is still in development, driving simulation modelling can be conducted using CAV logic but any prediction of future traffic conditions may be used to assess potential scenarios on what may happen once the CAVs will penetrate the market and will be widespread on the road network. 

In this view, the 2022 HCM [2] presented the adjustment factors to modify the core methods of capacity estimation under different proportions of CAVs equipped with the cooperative adaptive cruise control system and enabled by vehicle-to-vehicle communication; these factors were derived from microsimulation under the assumption that all communication elements were working with a high degree of reliability. Based on the above, the 2022 HCM [2] was used in this research as an alternative source of target values of capacity since the vehicular fleets incrementally transitioning towards a fully CAV fleet are not yet observable in the field. The capacity adjustment factors for CAVs for roundabouts [2] are the only methodological support that responds to the actual need for the transportation authorities and companies to assess the potential of the cooperative driving in order to improve the gap usage and to provide the long-range effects on roads’ throughput. 

Starting from the geometry and traffic patterns observed in the field on existing roundabouts, “what-if” scenarios were conceptualised and analysed to assess the assumption-based behaviour of CAVs mixed with HDVs and to evaluate the effects of the progressive introduction of CAVs in traffic. Based on two case studies of an existing single-lane roundabout and a two-lane roundabout, two entry mechanisms (i.e., the entry mechanism 1 where the one-lane entry is conflicted by one circulating lane, and the entry mechanism 2 where the left entry lane in the two-lane entry is conflicted by two circulating lanes) were examined. The roundabouts in Figure 3a and Figure 4a were selected as case studies since they are representative examples of a single-lane roundabout and a large diameter (two-lane) roundabout, both appropriate to examine the entry mechanisms 1 and 2 above [1,2]. The selected roundabouts are installed in suburban areas similar to the urbanistic point of view where the availability of space in the transition between the countryside and the city, and the flat terrain, have favoured the radial alignment of the four legs at about a 90-degree angle; similar deflections through the roundabouts were also measured in the field. It should be noted that the priority rule to vehicles circulating around the central island and the curved trajectories on roundabouts may make the decision making by vehicles instead of human drivers even more challenging than at stop-controlled or signalised intersections. However, the geometry where the curved trajectories are predominant, enables traffic to enter, to circulate and to exit at speeds complying with the speed limit. The geometric design also enables appropriate sight distances to ensure drivers to perceive and react to the presence of conflicting vehicles. Surveys showed that both roundabouts had relatively balanced traffic flows from all the legs; low likelihood of daytime and night-time pedestrian activity was also observed since the intersections are far enough from the residential and commercial area of their respective cities. The size of the circulatory roadway and the entry (or exit) geometric design on the single-lane roundabout in Figure 3a cannot admit lane changing on them. In turn, the size of the circulatory roadway and the entry (or exit) geometric design of the two-lane roundabout in Figure 4a can admit lane changing (i.e., two vehicles can advance side by side or, depending on the availability of acceptable gaps, lane change may be possible). Again, the need for two headways equally probable in the inner lane and in the outer lane on the circulatory roadway to enter the two-lane roundabout makes the entry mechanism 2 more challenging than the mechanism 1 on the single-lane roundabout (see e.g., [1]).

For the specific consequences on the performances of the considered entry mechanisms, this paper focused on the simulation of the CAVs’ operation rather than the Avs’ counterpart. The CAV-adjusted capacity curves from the HCM [2] were used as capacity target values to who’s the simulated capacities were compared. After successful completion of the fine-tuning process of the behavioural parameters of Aimsun Next [25], the results highlighted that higher penetration rates returned higher capacity values (see Figure 9a). The statistical tests confirmed that the difference between the means of the data subsets of CAV-adjusted and simulated data by scenario was due to random chance. 

The “what-if” scenarios with CAVs allowed us to clarify the contribution of the geometric shape of each roundabout (in terms of size of the outer diameter, number of entry and exit lanes, and number of lanes on the circulatory roadway) on their respective operational performances. However, although trajectory planning for CAVs has the potential to improve efficiency and vehicle fuel economy in traffic systems, the management and control of CAVs on curvilinear trajectories still require further study (see e.g., [76]).

Operating conditions at capacity were simulated in Aimsun Next for the two mechanisms of entry which we examined since the presence of CAVs is expected to create an incentive to operate the entry mechanisms at a high level of utilization. The higher the proportion of the CAVs in traffic, the more frequently the benefits of connectivity can be obtained, since more vehicles can accept smaller gaps safely. There is also consistency with the research findings that the literature presents, since the proportion of the traffic that is made of CAVs influenced the increases in capacity (e.g., [51]). To take some examples, if there are 40% of CAVs (i.e., scenario 2), compared to the baseline scenario the entry capacity increased by about 15.00% in the case of the entry mechanism 1 and about 17.00% in the case of the entry mechanism 2 (see Figure 9a). Similarly, in scenario 3 with the CAV penetration rate of 60%, a significant reduction of the delay and travel time values was achieved for the single-lane entry with percentage differences up to about 13.50% and 11.00%, respectively (see Figure 9b,c). There was also a significant reduction of the delay and travel time values for the entry mechanism 2 with percentage differences about of 18.78% and 16.68%, respectively (see Figure 9b,c). However, the slightly higher percentage reductions achieved for the entry mechanism 2 compared to the entry mechanism 1 are depending on the more assertive driving required to enter the two-lane roundabout, where the entering vehicle is conflicted by two traffic streams on the circulatory roadway. Consistently with the literature (e.g., [77]) the percentage differences of delay and travel time tend to be stabilized transitioning towards a fully CAV fleet (see Figure 9b,c). Despite similar trends in both entry mechanisms, however, further layouts of roundabouts should be studied to better understand how the roundabout design standards should be updated in order to improve the existing road network in terms of traffic efficiency and to make it appropriate for the progressive introduction of CAVs in traffic. 

Although the progressive transition to an all-CAV fleet showed clear benefits on the efficiency of the entry mechanisms examined here, the traffic scenarios executed in Aimsun Next [25] under CAV logic should be considered as assumption-based examples of what may happen with the presence of CAVs in traffic rather than being considered as the definitive word on what will happen with fully operational CAVs on the road net-work. Thus, we are aware that a definitive conclusion on the optimum penetration rate cannot be reached due to the lack of a high level of CAVs in the real world that could confirm (or not) the assumptions underlying this study.

## 6. Conclusions

Although the combination of the connectivity and automation technologies with the transport system is expected to provide increases in road safety, traffic efficiency and energy savings within the near future, there are still many open questions regarding how CAVs in traffic may affect the roadway performance and what methods should be used to assess the desired benefits of greater proportions of CAVs on the road network. 

Roundabouts are an example of sustainable and resilient intersections because they do not have traffic lights where cars stop or idle, they moderate vehicular speeds and can reduce delay and exhaust emissions; differently from signalised intersections, roundabouts keep functioning after storms without needing electricity for traffic lights [78]. Despite this, there is still the need to study how autonomous vehicles can operate in a complex and dynamic environment as roundabouts where the cooperation with other participants in traffic must take account of the curvilinear nature of the geometric design and the traffic patterns that may occur. In a transient state of CAV implementation, there is the need to study the key operational issues when HDVs and CAVs are mixed in the same road or intersection. The question arises as to what the prospects about the performance of CAVs on roundabouts are where the interpretation of the intentions of the other vehicles may be much more challenging due to the curvilinear trajectories experienced through the roundabouts and the priority rule to circulating traffic. The lack of field data about CAVs on roundabouts still requires trying further assumptions when modelling situation-awareness driving behaviour and the impacts on roundabout performance; however, the resulting projections can vary from cautious to optimistic.

The field observation on suburban roundabouts were carried out in order to start a reflection on the functional scope of different entry lane types and to explore two different entry mechanisms with a one-lane entry conflicted by one circulating lane on the single-lane roundabout in Figure 3a and the left entry lane of a two-lane entry conflicted by two circulating lanes on the two-lane roundabout in Figure 4a. Differently from the single-lane roundabout, the chance of lane changing can be admitted in the two-lane layout where the need of two headways equally probable in the inner lane and in the outer lane on the circulatory roadway makes the entry mechanism more challenging than the single-lane counterpart. 

Capacity calculations were based on the capacity models and the adjustment factors for the CAVs proposed by the 2022 HCM for roundabouts [2]. The comparison between the CAV-adjusted capacity curves and simulated capacities helped us to endorse hypothesis on the model parameters of Aimsun Next which mostly affect the CAVs’ ability to in-crease the roundabouts’ throughput. The fine-tuning model parameters of Aimsun Next improved the agreement of the simulated data with the capacity target values calculated under different proportions of CAVs in traffic. 

The “what-if” scenarios with CAVs built in Aimsun Next allowed us to evaluate the effect of the geometric shape of each roundabout in terms of size, number of entry and exit lanes, and number of lanes on the circulatory roadway on their respective efficiency. The results returned in Aimsun Next highlight how the introduction of CAVs into traffic impacts the operational performances on roundabouts and provided a synthetic insight on the potential ability of CAVs and their long-range effects on the roundabouts’ operational performance. According to the results, the performance indicators such as capacity, delay and travel time gradually improved with the increase of the selected penetration rates of CAVs. However, the slightly higher percentage reductions achieved for the two-lane roundabout compared to the single-lane roundabout are depending on the more assertive driving required to enter the two-lane roundabout where the entering traffic is conflicted by two traffic streams on the circulatory roadway. The same values of gap acceptance will be decreased based on the characteristics that CAVs will have.

It should be noted that many of the considerations we made resulted from coherent assumptions based on the current knowledge of the research area, however, without the evidence that would make them acceptable and then generalizable. Although the progressive transition to an all-CAV fleet showed clear benefits on the efficiency of the entry mechanisms examined here, the traffic scenarios executed in Aimsun Next under CAV logic should be considered as assumption-based examples of what may happen with the presence of CAVs in traffic rather than being considered as the definitive word on what will happen with fully operational CAVs on the road network. Thus, the what-if scenarios provided a way to analyse the effects of CAVs on roundabouts although great variability in several factors exists. We are also aware that a definitive conclusion on the optimum penetration rate cannot be reached due to the lack of a high level of CAVs in the real world that could confirm the assumptions underlying this study. 

It should be noted that only the 2022 edition of the Highway Capacity Manual has provided for the first time capacity-adjustment factors for roundabouts to account for the presence of CAVs in the traffic stream. Despite the need for comparison with other methods or models, the methodology provided by the 2022 HCM is the only reference in the literature that can be employed to account for CAVs’ potential ability to increase (or not) existing roadways’ throughput. There is the need to explore a wider range of study cases and traffic demand patterns to investigate further correlations between the roundabout geometric design and the traffic patterns with CAVs, and to better understand how to improve roundabout design standards to upgrade the existing road infrastructures and equip them with the technologies that enable smart mobility. There is also the need for road management to be prepared for a future that may be sooner than expected. Thus, future research is also required about traffic safety and environmental impacts, as well as the use of other measures or methodological approaches that should be developed to assess the level-of-service on the road infrastructures as the CAVs will fully penetrate the market.

## Figures and Tables

**Table 1:** Capacity adjustment factors by penetration rate of CAVs for roundabouts. Source: Own research based on data presented in [2].

Penetration Rate of CAVs (%)	Capacity Adjustment Factors			
	Entry Mechanism 1 ^1^		Entry Mechanism 2 ^2^	
	*f_A_*	*f_B_*	*f_A_*	*f_B_*
0	1.00	1.00	1.00	1.00
20	1.05	0.99	1.03	0.99
40	1.12	0.97	1.08	0.96
60	1.22	0.94	1.18	0.92
80	1.29	0.90	1.28	0.89
100	1.35	0.85	1.38	0.85

^1^ entry mechanism 1: one-lane entry conflicted by one circulating lane; ^2^ entry mechanism 2: left entry lane of the two-lane entry conflicted by two circulating lanes.

**Table 2:** Vehicle modelling parameters of Aimsun Next for the study roundabouts. Own research based on the default data presented in [25].

Model Parameters	Default Values				Fine-Tuned Values	
	Mean	Dev	Min	Max	HDVs	CAVs
Max desired speed [km/h]	110	10	80	150	50 ^1^	50 ^1^
Vehicle length [m]	4.00	0.50	3.50	4.50	4.00	4.00
Vehicle width [m]	2.00	0.00	2.00	2.00	2.00	2.00
Clearance [m]	1.00	0.30	0.50	1.50	1.00	1.00
Lateral clearance [m]	0.10	0.30	0.10	0.10	0.50	0.50
Reaction time ^2^ [s]	0.80	-	0.80	0.80	0.86 (0.95) ^3^	0.63
Gap [s]	0.00	0.00	0.00	0.00	1.58 (1.33) ^3^	0.00
Speed limit acceptance	1.10	0.10	0.90	1.30	1.00 (0.97) ^3^	1.00
Maximum acceleration [m/s^2^]	3.00	0.20	2.60	3.40	4.00	4.00
Normal deceleration [m/s^2^]	4.00	0.25	3.50	4.50	4.00	4.00
Maximum deceleration [m/s^2^]	6.00	0.50	5.00	7.00	6.00	6.00
Safety Margin Factor	1.00	0.00	1.00	1.00	1.00	0.50
Sensitivity Factor	1.00	0.00	1.00	1.00	*na* ^4^	1.00 (0.60) ^3^
Cooperate in creating a gap ^5^	-	-	-	-	*na* ^4^	0.50 ^7^
Imprudent lane-changing ^6^	-	-	-	-	*na* ^4^	yes ^7^
Headway aggressiveness	0.00	0.00	−1.00	1.00	*na* ^4^	0.00

^1^ The value corresponds to the actual limit imposed on the examined roundabouts; ^2^ value without deviation so that minimum and maximum values are constant values; ^3^ the values within round brackets concern the two-lane roundabouts; ^4^ *na* stands for not applicable; ^5^ this parameter denotes the percentage of upstream vehicles cooperating to create a gap for a vehicle that tries to change lane, and it can be flagged or not; ^6^ this parameter defines whether a CAV will still change lane after assessing an unsafe gap and it can be flagged or not; ^7^ this parameter was flagged just for the two-lane roundabouts.

**Table 3:** Summary statistics for the CAV-adjusted and simulated capacity values for different penetration rates of CAVs on the two-lane roundabout.

Market Penetration Rates of CAVs [%]						
Capacity (pc/h)	0	20	40	60	80	100
*μ*_1_^1^ (s.e.)	774.46 (57.97)	745.93 (56.58)	786.75 (58.35)	881.15 (62.69)	980.67 (67.64)	1067.83 (71.40)
*μ*_2_^1^ (s.e.)	731.12 (67.97)	738.00 (66.28)	799.77 (68.27)	887.44 (72.47)	1001.66 (78.52)	1107.11 (85.92)
95% c.i. ^2^	(−135.3, 221.9)	(−165.9, 181.8)	(−192.2, 166.1)	(−197.4, 184.8)	(−227.7, 185.7)	(−262.1, 183.5)
*t*-statistic ^3^	0.50	0.10	0.15	0.10	0.20	0.35
*t*-critical	2.0003	1.995	1.995	1.994	1.995	1.995
*p*(α)-value ^4^	0.63	0.93	0.88	0.95	0.84	0.73
*F*-statistic ^5^	1.37	1.37	1.37	1.34	1.35	1.45
*F*-critical ^6^	1.822	1.757	1.757	1.757	1.757	1.757
*F*-prob ^7^	0.38	0.35	0.36	0.40	0.38	0.28
RMSNE [24]	0.13	0.09	0.07	0.07	0.06	0.07
GEH (%) [24]	91	100	100	97	94	92

^1^ *µ*_1_ and *µ*_2_ are the means of the samples of equal size, while s.e. stands for the standard error for the mean; ^2^ 95% confidence interval for difference in means; ^3^ *t*-statistic from the *t*-test on N = 70 degrees of freedom at the significance level α = 0.05; ^4^ *p*(α)-value is the probability under null hypothesis of equal means at the significance level α = 0.05; ^5^ *F*-statistic from the two-tailed *F*-test: the hypothesis that the two variances were equal is rejected if *F*-statistic > *F*-critical value; ^6^ *F*-critical value of the *F* distribution is $F_{\alpha ,\;N_{1}-1,N_{2}-1}$ with *N*_1_−1 and *N*_2_−1 degrees of freedom and significance level of α = 0.05; ^7^ F-prob means the probability under null hypothesis of equal variances.

## Data Availability

Data are available after kind request to the corresponding author.
